# Polyunsaturated Fatty Acid Dietary Supplementation Induces Lipid Peroxidation in Normal Dogs

**DOI:** 10.4061/2010/619083

**Published:** 2010-06-27

**Authors:** John M. Walters, Timothy B. Hackett, Gregory K. Ogilvie, Martin J. Fettman

**Affiliations:** Department of Clinical Sciences, Colorado State University, 300 West Drake Road, Fort Collins, CO 80523, USA

## Abstract

Polyunsaturated fatty acids (PUFAs) have anti-inflammatory effects at low concentrations; however increased dietary consumption may conversely increase susceptibility to oxidation by free radicals. The objective of this study was to determine the effects of PUFAs on selective oxidative injury and inflammatory biomarkers in canine urine and serum. Dogs (*n* = 54) consumed a diet supplemented with 0.5% conjugated linoleic acid/dry matter, 1.0% conjugated linoleic acid/dry matter, or 200 mg/kg docosahexaenoic acid/eicosapentaenoic acid for 21 days. All dogs exhibited significantly increased plasma PUFA concentrations. All dogs had significant elevations in urinary F_2*a*_ isoprostane concentration, though dogs consuming a diet containing 1.0% conjugated linoleic acid/dry matter had the highest increase (*P* = .0052). Reduced glutathione concentrations within erythrocytes decreased significantly in all three dietary treatment groups (*P* = .0108). Treatment with diets containing 1.0% conjugated linoleic acid/dry matter resulted in the greatest increase in oxidant injury. Caution should be exercised when supplementing PUFAs as some types may increase oxidation.

## 1. Introduction

PUFAs are known to exert protective cellular effects against injury, though the exact mechanism of protection is unknown [1]. PUFAs are typically consumed in low quantities. Studies evaluating PUFA dietary supplementation have had conflicting results [2]. Supplementation in excess of normal dietary levels can result in a paradoxical increase in susceptibility to free radical oxidant injury, resulting from a combination of both relative increase in PUFA concentration and relative decrease in antioxidant content [2]. Reactive oxygen species react with the double bond of these unsaturated lipids resulting in lipid peroxidation, and increased risk of oxidative stress [3]. 

The objective of this study was to determine the effects of 21 days of PUFA dietary supplementation on plasma PUFA levels and select oxidative injury and inflammatory biomarkers in urine and serum in healthy dogs. We hypothesized that PUFA supplementation would augment plasma PUFA levels and that augmentation may alter the REDOX status of these healthy dogs.

## 2. Materials and Methods

The Institutional Animal Care and Use Committee approved all procedures prior to conducting this research. Healthy dogs were recruited from the students of the Colorado State University College of Veterinary Medicine. Health was assessed by physical examination, complete blood count, serum biochemical panel, and routine urinalysis. Signalment and weight were recorded. Once enrolled, dogs underwent additional blood and urine testing to evaluate baseline PUFA concentration and markers of oxidant injury and inflammation. Dogs were randomly assigned to one of three polyunsaturated fatty acid treatment groups in which they consumed a diet supplemented with a capsule containing 0.5% and 1.0% conjugated linoleic acid/dry matter(Conjugated linoleic acid, Experimental and Applied Science, Golden, CO.; Composition verified by Integrated Biomolecule Corporation, Tucson, AZ.), or 200 mg/kg body weight docosahexaenoic acid/eicosapentaenoic acid (Omega Pet, NBF-LANES srl, Milano, Italy.) for 21 days. In addition to PUFA supplementation, dogs were fed their usual diets by the owners with no changes in composition or quantity. 

At 0 and 21 days, blood and urine were evaluated for PUFA concentration and markers of oxidant injury and inflammation. Twenty-four mLs of blood was collected by jugular venipuncture into collection tubes containing EDTA, scavenger, or no additive. Whole blood samples in EDTA were centrifuged at 2000 g for 15 minutes. Plasma was then decanted and frozen at −70°C until analysis. Plasma fatty acids were measured by gas chromatography [4] (HP Gas Chromatograph, Model 5980 series II, San Fernando, CA.). Specific analytes included CLA (18 : 2 *c*9, *t*11 and 18 : 2, *t*10, *c*12), DHA (C22 : 6n-3), and EPA (C20 : 5n-3). Small aliquots of whole blood were placed into cryovials(Nalgene Cryoware, Nalge Company, Rochester, NY.) containing either no additive or 10% 1-methyl-2-vinylpyridinium trifluoromethanesulfonate scavenger for subsequent erythrocyte reduced and oxidized glutathione (GSH:GSSG) analysis. These samples were immediately flash-frozen in liquid nitrogen and stored at −70°C. Erythrocyte GSH:GSSG concentration was performed using a commercially available test kit (GSH/GSSG-412 Assay System, Oxis Research, Portland OR). The remaining whole blood samples without additive were allowed to clot and then centrifuged at 2000 g for 15 minutes. Serum was then decanted and frozen at −70°C until analysis. Serum C-reactive protein (CRP) was measured by spectrophotometry [5]. Urine was collected by voluntary void or cystocentesis and stored at −70°C. Urine 8-epi-prostaglandin F_2*α*_ (iPF_2*α*_-III) by commercial ELISA. ( 8-isoprostane ELISA, Genox Corporation, Baltimore, MD.).

## 3. Statistical Methods

A modified Kolmogorov-Smirnov test was used to assess normality of data distribution. Continuous data were reported as mean and standard deviation. Bartlett's test of homogeneity was used to assess equality of variances for analysis of variance (ANOVA). An ANOVA for treatment effects, with repeated measures for time effects, followed by Fisher's least significant difference test, was used to identify individual group/time differences. Associations between continuous variables were assessed by linear regression analysis. Fisher's “*r* to *z*” test was used to evaluate coefficients. Results were considered significant at *P* < .05.

## 4. Results

All dogs were judged to be healthy based on normal physical examination, complete blood count, serum biochemical panel, and routine urinalysis prior to inclusion. Mean age of dogs was 6 years and ranged from 1 to 10 years. Mean weight of dogs was 25.5 kg (SD: 9.54 kg). Twenty-nine males and 25 females were included: ten males and 9 females in the 0.5% conjugated linoleic acid/dry matter group, 9 males and 8 females in the 1.0% conjugated linoleic acid/dry matter group, and 10 males and 8 females in the 200 mg/kg docosahexaenoic acid/eicosapentaenoic acid group. Dogs did not receive any anti-inflammatory drugs or nutrient supplements during the study period.

Continuous data are summarized in Table 1. Plasma PUFA concentrations increased significantly following supplementation in all treatment groups. No linear associations were detected between elevation in plasma PUFA concentration and markers of oxidative damage. Pretreatment erythrocyte GSH concentrations were significantly higher than GSH posttreatment concentrations in all groups (*P* = .0108), but no significant individual treatment effects were detected. No change was seen in GSH:GSSG ratios. There were no significant changes in CRP concentrations with respect to treatment or time effects (*P* = .6102).

Urinary isoprostanes (iPF_2*α*_-III) were elevated in dogs consuming 1.0% conjugated linoleic acid/dry matter following 21 days of treatment (*P* = .0102). In addition, post-treatment levels of iPF_2*α*_-III were significantly higher in this group than both the 0.5% conjugated linoleic acid/dry matter (*P* = .0052) and 200 mg/kg docosahexaenoic acid/eicosapentaenoic acid (*P* = .004) treatment groups.

## 5. Discussion

In this study, PUFA dietary supplementation in healthy dogs over a 21-day period resulted in increased measured plasma fatty acid concentrations. In addition, we documented elevation in lipid peroxidation with PUFA dietary supplementation as illustrated by decreased GSH and elevated urinary isoprostanes. Presence of diminished quantities of GSH implies consumption via oxidant injury. Elevation in urinary isoprostanes is consistent with lipid peroxidation, as these compounds result from the reaction of arachidonic acid contained within cellular membranes and reactive oxidant species [6]. However, the levels of isoprostanes found in this paper have not been correlated with histologic or metabolic disturbance. In addition, corresponding markers of inflammation (CRP) were not elevated. In this population, we documented a diminished antioxidant status. Despite this alteration in baseline antioxidant status, no change in markers of inflammation was anticipated in the absence of systemic insult, such as that due to disease or trauma. Furthermore, 21 days of supplementation may not be sufficient to detect this change. In a study of normal dogs undergoing a combination of EPA and DHA dietary supplementation over 12 weeks, decreased inflammatory cytokine activity was documented [7]. Further support can be found in a recent study where combined EPA and DHA dietary supplementation over 56 days also augmented plasma PUFA concentrations but decreased concentration of synovial degradative enzymes in dogs with inflammatory joint disease [8]. Although the mechanism of action of these supplements on joint health was unknown, the authors theorized that the beneficial effects seen with supplementation were due to diminished release of inflammatory cytokines.

Study limitations are related to the population studied. Dogs were of various breeds and ages and underwent unspecified management routines. Diet was also not controlled. Dietary requirement for vitamin E is closely related to PUFA consumption as sequestration of Vitamin E allows maintenance of antioxidant stability, with the ratio of Vitamin E/PUFA recommended in human diets is 0.4 mg/gram [9]. As this study incorporated healthy dogs on various diets, the vitamin E to PUFA ratio was not evaluated. However, the population of dogs evaluated was designed to represent a subpopulation of typical healthy dogs maintained in the home. This population is the target for evaluation of dietary supplements given in addition to a dog's routine diet. Dietary supplements are often given without regard for other dietary intake and the effect of supplementation under these conditions was the primary interest. To minimize variability and ensure appropriate comparisons, dogs acted as their own control with plasma, serum, and urine measurements performed prior to and following dietary supplementation.

 Due to conflicting reports in efficacy of PUFA dietary supplementation in dogs, this area of nutrition research requires further study. Healthy dogs supplemented with CLA in this report experienced the highest increase in markers of lipid peroxidation and this increase was dose dependent. Caution should be exercised when supplementing commercial canine diets with PUFAs as some types may increase the risk of lipid peroxidation and oxidant injury. Owners should be advised prior to empirical supplementation of fatty acids.

## Figures and Tables

**Table 1:** Summary of effect of PUFA dietary supplementation on PUFA levels and indices of oxidant injury and inflammation. Results reported as mean and standard deviation. The symbol  ∗ denotes significant change between Day 0 and Day 21 measurements and superscript letters indicate significant differences between treatment groups. CLA Conjugated linoleic acid DHA docosahexaenoic acid EPA eicosapentaenoic acid *i* *P* *F*_2*α*_-*I* *I* *I* urine 8-epi-prostaglandin *F*_2*α*_ CRP C reactive protein.

Diet Treatment	0.5% CLA		1.0% CLA		DHA/EPA	
Timepoint	DAY 0	DAY 21	DAY 0	DAY 21	DAY 0	DAY 21

CLA (18 : 2 *c*9, *t*11) mg/dL	2.39 ± 3.1	2.49 ± 1.6*	3.01 ± 3.0	3.34 ± 2.6*	1.70 ± 2.1	1.83 ± 2.6*
CLA (18 : 2, *t*10, *c*12) mg/dL	1.61 ± 3.6	2.65 ± 2.0*	2.25 ± 3.5	4.16 ± 4.0*	0.92 ± 1.6	1.01 ± 1.4*
DHA (C22 : 6n-3) mg/dL	7.34 ± 3.6	9.88 ± 8.6*	10.10 ± 8.3	12.74 ± 9.6*	11.81 ± 9.8	19.61 ± 8.8*
EPA (C20 : 5n-3) mg/dL	2.31 ± 1.5	2.61 ± 1.7*	2.28 ± 1.0	2.90 ± 2.1*	3.09 ± 2.6	9.44 ± 5.6*
GSH *μ*M/L	881.9 ± 240.3	747.5 ± 339.1*	1045.4 ± 429.3	617.0 ± 418.3*	814.9 ± 277.1	836.0 ± 225.1*
GSSG *μ* *M*/L	46.3 ± 51.6	40.0 ± 61.7	50.5 ± 44.5	23.2 ± 24.0	35.4 ± 24.2	40.3 ± 29.0
GSH:GSSG	52.6 ± 75.1	77.2 ± 100.5	52.1 ± 60.9	45.8 ± 54.0	52.0 ± 80.3	58.6 ± 85.1
iPF_2*α*_-III pg/mg creatinine	901.6 ± 376.7	1169.6 ± 484.3^∗a^	1144.4 ± 484.6	1673.8 ± 815.5^∗b^	819.4 ± 412.7	995.0 ± 440.4^∗c^
CRP mg/dL	0.51 ± 0.07	0.51 ± 0.77	0.49 ± 0.03	0.52 ± 0.08	0.51 ± 0.07	0.49 ± 0.03

^a^Conjugated linoleic acid, Experimental and Applied Science, Golden, CO.

^b^Conjugated linoleic acid purity and composition verified by Integrated Biomolecule Corporation, Tucson, AZ.

^c^Omega Pet, NBF-LANES srl, Milano, Italy.

## Abbreviations

ANOVA: analysis of variance
CLA: conjugated linoleic acid
CRP: C-reactive protein
DHA: docosahexaenoic acid
EDTA: ethylenediaminetetraacetic acid
EPA: eicosapentaenoic acid
GSH: reduced glutathione
GSSG: oxidized glutathione
iPF_2*α*_-III: 8-epi-prostaglandin F_2*α*_
PUFA: polyunsaturated fatty acids
REDOX: reduction-oxidation
